# Readiness of Medical Students to Care for Diverse Patients: A Validated Assessment of Cross-Cultural Preparedness, Skills, and Curriculum

**DOI:** 10.1089/heq.2023.0142

**Published:** 2023-09-13

**Authors:** Andrew D.P. Prince, Alexander R. Green, David J. Brown, Michael J. Brenner

**Affiliations:** ^1^ Department of Otolaryngology–Head and Neck Surgery, University of Michigan, Ann Arbor, Michigan, USA.; ^2^ Division of General Internal Medicine, Massachusetts General Hospital, Boston, Massachusetts, USA.; ^3^ Office of Medical Student Education, University of Michigan Medical School, Ann Arbor, Michigan, USA.; ^4^ Office for Health Equity and Inclusion, Ann Arbor, Michigan, USA.

**Keywords:** diversity, medical education, cultural competency, health disparities, social inequities

## Abstract

**Introduction:**

Effective cross-cultural care is foundational for mitigating health inequities and providing high-quality care to diverse populations. However, medical school teaching practices vary widely, and learners have limited opportunities to develop these critical skills. To understand the current state of cross-cultural education and to identify potential opportunities for improvement, we disseminated a validated survey instrument among medical students at a single institution.

**Methods:**

Learners across 4 years of medical school participated in the cross-cultural care assessment, using a tool previously validated with resident physicians and modified for medical students. The survey assessed medical student perspectives on (1) preparedness, (2) skillfulness, and (3) educational curriculum and learning environment. Cross-sectional data were analyzed by class year, comparing trends between school years.

**Results:**

Of 700 possible survey responses, we received 260 (37% response rate). Fourth-year students had significantly higher scores than first-year students (*p*<0.05) for 7 of 12 preparedness items and 4 of 9 skillfulness items. Less than 50% of students indicated readiness to deliver cross-cultural care by their fourth year in 9 of 12 preparedness items and 6 of 9 skillfulness items. Respondents identified inadequate cross-cultural education as the primary barrier.

**Discussion:**

Medical students reported a lack of readiness to provide cross-cultural care, with self-assessed deficiencies persisting through the fourth year of medical school. Medical educators can use data from the cross-cultural care survey to longitudinally assess students and enhance curricular exposures where deficiencies exist. Optimizing cross-cultural education has the potential to improve the learning environment and overall patient care.

## Introduction

The COVID-19 pandemic has laid bare racial, ethnic, and other sociodemographic health inequities. Improving cross-cultural care is a necessary step in understanding and addressing the sociocultural factors^1^ and structural inequities^2^ that drive disparate outcomes. Although cross-cultural education is an integral aspect of undergraduate medical curricula, implementation and assessment remains inconsistent.^3,4^ As a result, unexplored and under-considered sociocultural factors in patient–physician communication can lead to unequitable care, poor adherence, mistrust, and worse health outcomes.^5,6^ Cross-cultural care training can empower health care professionals to recognize a patient's health beliefs, values, and preferences, thereby improving quality of care and mitigating disparities.^7–9^ However, such benefits will only be realized if medical education can support development of these skills.^10^

Cross-cultural care curricula are required by the Accreditation Council for Graduate Medical Education, in recognition of the need to reduce bias and provide safe, high-quality care to diverse patients.^11^ A significant barrier to progress is the gap in knowledge around current practice and areas of deficiency. Prior studies have documented variable content, teaching, timing, and assessment of learners.^12–15^ Thus, although significant efforts are directed at improving cross-cultural competency, relatively few learners are likely to graduate medical school equipped to provide patient-centered care across diverse cross-cultural contexts.^13,16,17^ For example, fewer than half of students at Harvard Medical School felt comprehensively prepared to deliver cross-cultural care.^18^ Thus, although cross-cultural care has the promise of improving outcomes, more purposeful efforts to teach and assess cross-cultural education are necessary.

To gain a deeper understanding of medical students' perspectives on cross-cultural care and education, we adapted and distributed the validated cross-cultural care survey (CCCS) for medical students at the University of Michigan Medical School. We assessed self-perceived skills and preparedness as well as the cultural climate and learning environment in medical students all 4 years. We did not implement a specific intervention as part of this study; rather, we used the CCCS to assess the existing curriculum, found on the University of Michigan Medical School website.^19^ We hypothesized that fourth-year students would have greater preparedness and skills than first-year students, years 2 and 3 were clinical. These data serve as a roadmap for curricular innovation, including opportunities for enhancing didactic and experiential learning.

## Methods

### Survey design and administration

We used the validated Internet-based survey, previously utilized at Harvard Medical School^18^ to investigate the preparedness and skillfulness of medical students at providing cross-cultural care. We also asked about the learning environment at the medical school and captured personal and professional characteristics. We obtained permission to adapt and use the modified CCCS for medical students^18^ and made minor modifications to ensure relevance of all questions to the institution.

The survey was conducted in accordance with the University of Michigan institutional procedures (IRB No. HUM00174766). We inserted the survey into QualtricsXM^®^ and distributed the survey, by e-mail listserve for years 1–4, in early February to all medical students. We chose to exclude dental and MD/PhD students from the current study due to differences in training schedule and curriculum. Participation in the online survey was voluntary for all students. To enhance our response rate, we sent out three reminder e-mails to students separated by 5 days.

### Variables

Two constructs related to readiness to provide cross-cultural care were measured: (1) preparedness to care for specific types of patients and (2) self-assessment of specific cross-cultural skills. We also included questions assessing the educational curriculum and climate around cross-cultural care.

To assess preparedness, medical students indicated how prepared they felt to care for a series of types of patients (a response of 1=Very Unprepared, 2=Somewhat Unprepared, 3=Somewhat Prepared, 4=Well Prepared, 5=Very Well Prepared). The list includes patients from cultures different from their own; patients with health beliefs at odds with Western medicine; patients with religious beliefs that might affect treatment; patients who were new immigrants to the United States; patients with limited English proficiency; lesbian, gay, bisexual, and transgender patients; patients with disabilities; and patients who use alternative or complementary medicines.

Students were asked to assess their skill level in performing tasks relevant to treating culturally diverse patients (a response of 1=Not at All Skillful to 5=Very Skillful). These include determining how to address patients from different cultures, assessing patients' understanding of their illness, identifying mistrust, negotiating treatment plans, assessing English proficiency, identifying relevant cultural and religious beliefs, understanding decision-making roles, working with interpreters, and counseling patients about their use of complementary or alternative medicine.

### Educational curriculum and climate

Respondents also responded to questions relating to how the educational curriculum incorporated cross-cultural learning and the overall climate for cross-cultural learning. To assess the curriculum, students were asked to specify the courses completed and how each class prepared them to interact with culturally diverse patients using the following rating scale (a response of 1=Not at All Useful, 2=Useful, 3=Very Useful, and 4=N/A).

To assess the educational climate, we asked medical students to rate “how much of a problem” (1=No Problem, 2=Small Problem, 3=Moderate Problem, and 4=Big Problem) the following has been during medical school: (1) lack of practical experience caring for diverse patients; (2) inadequate cross-cultural care training; (3) absence of good role models or mentors in cross-cultural care; (4) dismissive attitudes about cross-cultural care among physicians; and (5) dismissive attitudes about cross-cultural care among fellow students. We also asked students to rate (1=Strongly Disagree, 2=Slightly Disagree, 3=Slightly Agree, 4=Strongly Agree) their level of agreement on the inclusion of cross-cultural care curricula into courses and clinical practice. Other questions assessed medical student demographic characteristics, including gender, race/ethnicity, language, and school year.

### Analyses

Chi-square analyses were used to analyze differences in preparedness and skillfulness by medical student year in the curriculum. All preparedness and skillfulness item responses were dichotomized into “Unprepared/Unskilled” (response of “1,” “2,:” or “3”) and “Prepared/Skilled” (response of “4” or “5”) and examined for change between year 1 and 4. Furthermore, responses about the extent of problems during medical training were dichotomized into the categories of “No Problem” (response of “1”) and “Problem” (responses of “2,” “3,” and “4”) and analyzed with Crosstab analyses. Demographics were examined using frequency analyses. *p*<0.05 was considered statistically significant for all analyses.

## Results

### Respondent characteristics

Of 700 possible survey responses across the medical school, we received 311 responses. Of those, we excluded 51 due to incomplete responses. After accounting for these exclusions, the final sample was 260 (response rate 37.1%).

Table 1 shows demographic characteristics for the medical students who participated in this study. There was a female predominance of respondents (female=61.1%; male=38.4%), especially in the first 2 years. The percentage of non-white respondents was higher in earlier class years (47% in year 1 vs. 22% in year 4). The percentage of white respondents was 64.7%, with the second largest group comprising Asians/Pacific Islanders (15.5%).

**Table 1.** Demographics of Medical Student Respondents to Cross-Cultural Care Survey

	% Of respondents				
	Year 1, ***N***=66	Year 2, ***N***=50	Year 3, ***N***=72	Year 4, ***N***=72	Year 1–4, ***N***=260
Gender					
Male	34.8	26	41.6	47.2	38.4
Female	65.2	74	58.4	52.8	61.1
Race/ethnicity					
White	53	59.2	66.2	77.8	64.7
Black	12.1	4.1	4.2	5.6	6.6
Asian or Pacific Islander	21.2	16.3	14.1	11	15.5
Native American/Alaskan Native	0	2	1.4	0	.8
Hispanic/Latino	6.1	4.1	4.2	2.8	4.2
Other	7.6	14.3	9.9	2.8	8.1

### Preparedness and skillfulness

Table 2 demonstrates the 12 measures of students' self-reported preparedness to provide cross-cultural care. Table 2 shows nine measures of students' self-reported skillfulness specific to cross-cultural care. Findings were ordered based on the absolute percentage difference in reports between students in the first year of medical school and those in the fourth year. Fewer than 50% of students felt prepared on 9 out of 12 preparedness items; and fewer than 50% of students reported skillfulness on 6 out of 9 items by their fourth year. Inadequate cross-cultural education was reported by medical students as the primary barrier (Table 3).

**Table 2.** Medical Student Readiness to Provide Cross-Cultural Care

	% Of respondents, ***N***=260				
	Year 1, ***N***=66	Year 2, ***N***=50	Year 3, ***N***=72	Year 4, ***N***=72	% Difference between year 1–4
Preparedness					
In general^*^	19.7	44	65.2	75	55.3
From cultures different from your own^*^	18.5	21.5	44.4	44.4	25.9
Who are transgender^*^	16.7	21.6	45.8	38.9	22.2
Who are gay, lesbian, or bisexual^*^	36.4	45.1	70.8	58.3	21.9
With limited English proficiency^*^	10.6	19.6	38.9	31.9	21.3
Who are persons with disabilities^*^	16.7	25.5	41.7	34.7	18
Who are members of racial and ethnic minorities^*^	42.4	43.1	65.3	59.7	17.3
With a distrust of the U.S. health care system	9.1	9.8	11.1	20.8	11.7
Who are new immigrants	13.6	9.8	20.8	22.2	8.6
Whose religious beliefs affect treatment	18.2	13.7	23.6	23.6	5.4
With health beliefs or practices at odds with Western medicine	10.6	11.8	12.5	13.9	3.3
Who use complimentary or alternative medicines	15.2	15.7	22.2	12.5	−2.7
Skillfulness					
Assessing the patient's understanding of the cause of their illness^*^	33.3	43.1	65.3	66.7	33.4
Negotiating with the patient about key aspects of the treatment plan^*^	12.1	25.5	40.3	43.1	31
Working effectively with a medical interpreter^*^	16.7	29.4	61.1	47.2	30.5
Taking a social history^*^	38.5	54.9	69.4	66.7	28.2
Identifying whether a patient is mistrustful of the health care system or physician	15.2	19.6	23.6	27.8	12.6
Determining how a patient wants to be addressed and interacted with	51.5	47.1	66.7	63.9	12.4
Identifying cultural (nonreligious) customs that might affect clinical care	7.7	13.7	12.5	18.1	10.4
Counseling patients about their use of complimentary or alternative medicine	10.6	13.7	13.9	20.8	10.2

Percentages shown reflect percent of students responding that they were well or very well prepared or skilled in the domains shown.

^*^ *p*<0.05 based on first year versus fourth year chi-square analysis.

**Table 3.** Medical Student Perceptions of Curriculum and Climate

	% Of respondents that disagree				
	Year 1, ***N***=66	Year 2, ***N***=50	Year 3, ***N***=72	Year 4, ***N***=72	All years, ***N***=260
Percentage of students who disagree with the statements					
The course directors at U of M incorporate cross-cultural issues into teaching	61.5	62.7	47.2	37.5	51.2
The clinical faculty at U of M incorporate cross-cultural issues into teaching	53.3	49	27.8	31.9	39.2
The leadership at U of M medical school makes learning about the care of culturally diverse patients a priority	49.2	54.9	38.9	33.3	43.1

	**% Of respondents**				
	**Year 1, *N*=66**	**Year 2, *N*=50**	**Year 3, *N*=72**	**Year 4, *N*=72**	**All years, *N*=260**
Percentage of students who believe the issues queried are problematic					
Inadequate cross-cultural training during medical school	90.9	86.3	87.5	84.7	87.4
Lack of practical experience caring for diverse patient populations	92.3	80.4	87.5	83	86.2
Absence of good role models or mentors for cross-cultural care among faculty	66.7	62	68.1	66.7	66.2
Dismissive attitudes about cross-cultural care among attending physicians	46.9	62	54.2	48.6	52.3
Dismissive attitudes about cross-cultural care among fellow students	44.6	47.1	51.4	41.7	46.2

The mean differences between first-year students and fourth-year students for the 12 reported preparedness items was 17.8% (range 2.7–55.3%). For the nine skill items, the mean difference was 19.6% (range 7.6–33.4%). Statistically significant differences (*p*<0.05) between year 1 and 4 students were found for 7 of 12 preparedness items. Exceptions involved lack of difference in caring for patients who either have distrust of the U.S. health system, use alternative medicines, harbor religious beliefs that affect treatment, have health beliefs at odds with Western medicine, or are new immigrants. The top 3 items that showed the greatest improvement across years were caring for patients either in general (+55.3%) or caring for patients with cultures different from one's own (+25.9%), or who are transgender (+22.2%).

For the nine skillfulness items, four items (assessing a patients understanding of their illness, negotiating key aspects of treatment, working with an interpreter, and taking a social history) showed statistically significant differences (*p*<0.05) between year 1 and 4. The three lowest item differences between first-year and fourth-year students included identifying religious beliefs that affect clinical care (7.6%), counseling on alternative medicine use (10.2%), and identifying cultural customs that affect clinical care (10.4%).

Of note, the third-year class reported the highest percentages, compared with the other classes, of preparedness in 6 of 12 categories. The third-year class also reported the highest percentages of skillfulness in three of nine categories. There were no significant differences between third-year and fourth-year reports of preparedness and skillfulness. Also, there were no changes in the preparedness or skillfulness items that were significantly different (between first-year and fourth-year) when first-year students were compared with third-year students.

### Learning environment

**FIG. 1.**
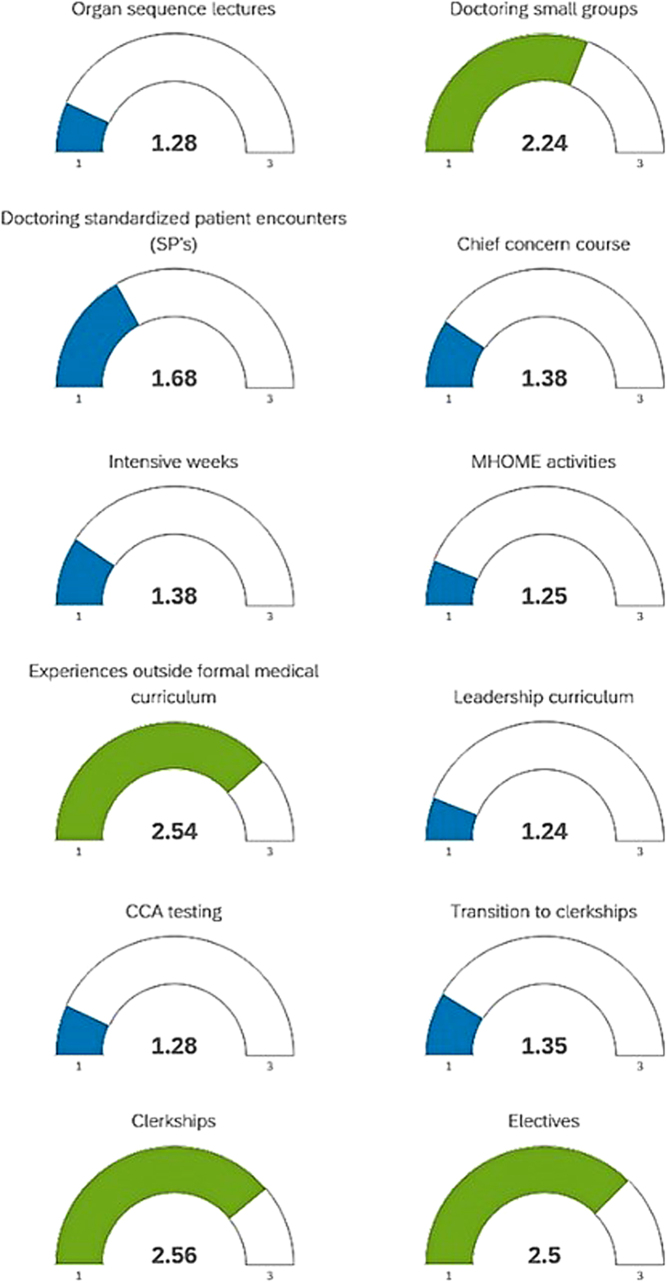
Student experiences useful for cross-cultural care learning. Reported mean usefulness of each course on a scale from 1 to 3 (1—not useful, 2—useful, 3—very useful) for all students.

Within the learning environment, only 3 medical courses of 12 queried were reported as useful for cross-cultural education; those deemed useful included doctoring small groups, clinical rotations, and clinical electives. Figure 1 shows reported mean usefulness of each course on a scale from 1 to 3 (1—not useful, 2—useful, 3—very useful) for all students. Among second-year students, 33.3% reported seeing greater than 20% racial and ethnic minority patients versus 47.22% and 37.5%, respectively, in years 3 and 4. We excluded first-year students from analyses of curricular offerings since exposure to curriculum was limited. Importantly, only 17.65% of second-year students reported seeing greater than 10% of patients with limited English proficiency and 20.8% and 22%, respectively, in years 3 and 4.

Respondents ascribed high importance to seeing a diverse mix of patients and highlighted the need for greater integration of cross-cultural education into existing curricular offerings. On a scale from 1 to 10, first year medical students responded with a mean of 9.1 on the importance of seeing a diverse racial/ethnic patient mix, versus 8.7, 8.5, and 7.8, in years 2, 3, and 4, respectively. Notably, 51.2% of all students disagreed that didactic course directors incorporate cross-cultural considerations into teaching and 39.3% of students disagreed with a statement that clinical faculty incorporate cross-cultural education into teaching (Table 3). Overall, 43% of students disagreed with the statement that University of Michigan makes learning about the care of culturally diverse patients a priority.

## Discussion

Despite growing awareness of the importance of cross-cultural care to medical education, there remains a gap between the aspiration of medical educators to instill such skills and the perceived readiness of learners to apply them. We found that our medical students do not feel adequately prepared to provide patient-centered care in a cross-cultural context, and prior work suggests that such lack of readiness persists among physicians-in-training and at later career stages.^18^ Many patient health outcomes are known to be heavily influenced by social determinants of health, and improving cross-cultural care is part of the larger moral imperative to widen our lane in medicine.^20^ Cross-cultural education equips doctors to care for individuals across diverse backgrounds, including race, ethnicity, gender orientation, socioeconomic status, cultural affiliation, and religious beliefs.^21^ The COVID-19 pandemic has underscored the importance of teaching students cross-cultural care to counter social and medical inequities.

Some of the available resources to improve cross-cultural care include checklists or assessment tools, which can guide medical educators in the design of courses that enable students to engage with patient diversity.^11^ However, the results of our survey reinforce prior observations noting a lack of robust and multifaceted experiences to promote cross-cultural care.^18^ An unanswered question is whether these shortfalls reflect inadequacy of the resources made available or inadequate integration of such resources into curricula.

Our survey data also reveal the ambivalence of students regarding efforts in this area. For example, 43% of students felt that learning about culturally diverse patients was not emphasized as a priority, and half felt that course directors did not consistently incorporate cross-cultural issues into teaching. This insight is crucial, as most medical students cited inadequate cross-cultural education and lack of experience as the foremost barriers to their maturation in this area. In addition, two-thirds of respondents noted a lack of role models. Half reported dismissive attitudes about cross-cultural care among attending physicians and fellow students. Such concerns were most marked in first- and second-year students, who were judging the learning environment based on lecture and small group dynamics, perhaps signaling a need for update of course materials and need to bring cross-cultural clinical experiences to earlier years.

The early effects of curricular improvements might be present within our data, especially during the clinical years, which include the second half of second year and all of third year. The third-year class demonstrated greater self-reported preparedness and skills in multiple items compared with the fourth-year class, but no measures were found significant (Table 2). When stratified by class, the doctoring course stood out, with 47% of third years finding it very useful compared with only 26.4% of fourth years. The course is actively overhauled each year and consists of small group discussions and experiential learning on topics, including alternative medicine, opioid pandemic, end of life discussions, shared decision making, and so forth. This result could be confounded by the low overall responses or the differences in student demographics.

Medical students' exposure to diverse cultural groups influenced their perception of readiness to engage in cross-cultural care. During medical training, students are socialized into a professional ethos that privileges Western medicine and normalizes institutional health care.^22^ This ethos and limited exposure to patients with other beliefs might partially account for low levels of preparedness to care for those who use alternative medicine or distrust the health system. For example, in our study, students reported seeing less than 20% of racial diverse patients and less than 10% of patients with limited English-speaking proficiency and were more likely to report lack of preparedness or skillfulness.

Similar to the Harvard medical student study,^18^ the LGBT and racial categories of patients received the highest levels of students' self-reported preparedness. This finding might reflect increasing social tolerance, or exposure, especially among the increasingly diverse medical student body. Nonetheless, unconscious bias can also adversely affect outcomes,^23^ and future study is necessary to verify whether self-reported perceptions will translate into equitable care and outcomes.

Our data corroborate the link between clinical experiences and readiness for cross-cultural care. Experiential learning is a critical aspect of cross-cultural education^24,25^; experiences in diverse communities afford breadth of understanding on the culture, needs, and challenges of marginalized groups. Such experiences can also instill a passion for change, encourage advocacy, and teach physicians the skills to counter the negative health outcomes imposed by poverty, inequality, and discrimination.^26^

Our results also highlight the importance of integrating cross-cultural education into early didactic years, which can be achieved through community experiences, service learning, and simulations. Course providers can explore ways to increase medical student exposure to immigrants, indigenous people, and other cross-cultural groups. Standardized patient simulations involving patients from different backgrounds and beliefs can offer a controlled environment for students to gain skills and confidence. Simulations can also teach shared decision making, which improves patient adherence, satisfaction, and outcomes.^27^

Community experiences can promote confidence in building rapport with patients who mistrust the health system or have different religious beliefs.^28^ Case-based learning can also help students understand how building trust between a patient and the medical team can improve care.^27^

Communication lies at the heart of cross-cultural care, which requires open dialog and cultural humility.^18^ In our survey, learners revealed limited growth from first to fourth year in three areas: identifying religious beliefs that affect clinical care, counseling on alternative medicine use, and identifying cultural customs that affect clinical care. Strategies that can address this limitation include practicing open-ended questions, shared decision making, and teach back methods. Cultural humility affords sensitivity to patients from different backgrounds and supports collaborative physician–patient decision making by diminishing power differentials and promoting trust; a stance of humility avoids the pitfall of assuming one has achieved cultural expertise or completed learning.^12^ The ideal atmosphere actively invites patient contributions and values patient experience and perspective alongside physician expertise.^29^

A few pedagogical approaches to developing patient–physician dialog and cultural humility have met with success: small-group discussions, personal journals, constructive professional role models from cultural groups, and videotaping with feedback.^12^ Community outreach clinics and service experiences can also promote cultural humility and advocacy to reduce health disparities.^24,26^

Clinical teachers have a vital role in modeling cross-cultural care,^22^ and doing so requires a willingness to go beyond the immediate biomedical considerations of patient care. In the busy clinical setting, cross-cultural education is often fragmented or omitted, perhaps partly because some clinical teachers themselves lack sufficient cross-cultural awareness.^30^ The limited literature on faculty development in this area suggest a paucity of faculty with expertise in designing and implementing cross-cultural curricular content^31^; comprehensive training of faculty and promoting mentorship across differences can provide a pathway for educators to offer structured cross-cultural experiences for learners.^32^

Our study has several limitations, primarily relating to potential sources of bias and limited generalizability. These include potential for selection bias, recall bias, or subjective responses. In addition, the cross-sectional design precluded assessment of individual classes over time. Also, we assessed students near the middle of the year, foregoing an assessment of students before entering medical school and omitting any additional improvement in the final portion of the fourth year. Although the survey instrument was validated, students may under- or overestimate their readiness.^16^ In the future will consider further tracking of students longitudinally over the course of their schooling and ideally into residency. Although the results presented in this study are consistent with prior work in resident physicians and at Harvard Medical School, a future multi-institutional rollout of the survey would improve generalizability.

Last, the study assessed current state without investigating specific interventions, but the work from this study was part of the impetus for ongoing innovations at our institutions in diverse areas, including the rollout of a health disparities curriculum, introduction of a history of racism course, and innovations around interprofessional education, all of which await formal study. The present study supports the feasibility of using the cross-cultural care validated survey as a diagnostic tool that can pinpoint areas for improvement and thereby assist in cultivating empathetic, and culturally aware clinicians who can provide safe, high-quality care for diverse patients.

## Conclusion

The CCCS, originally developed for assessment of physicians-in-training during residency, is a validated tool that can identify areas where medical school curricula can be improved. At University of Michigan, medical students perceived limited preparedness and skillfulness in care for culturally diverse patients, most marked in early years, but persisting through the fourth year of medical school. Respondents also highlighted limitations in cross-cultural curriculum and climate, suggesting a potential role for moving some cross-cultural education upstream to ensure meaningful exposures both early in medical school and sustained across the continuum of clinical experiences. The CCCS can guide curricular innovation in cross-cultural care through experiential learning, simulation, discussion, and practice. Finding from this survey provided an impetus to expand our own offerings and will hopefully also spur medical educators to use this tool to identify opportunities to improve cross-cultural education at their own institutions.

## Abbreviations Used

CCCS: cross-cultural care survey
LGBT: lesbian, gay, bisexual, transgender
